# Fine-Mapping and Protective Analysis of Immunodominant Linear B-Cell Epitopes of FimA Antigen of Klebsiella Pneumoniae

**DOI:** 10.3390/vaccines14040347

**Published:** 2026-04-15

**Authors:** Pengju Yan, Longlong Chen, Guangyang Ming, Zhifu Chen, Qiang Gou, Yue Yuan, Haiming Jing, Ping Luo, Jinyong Zhang, Zhuo Zhao

**Affiliations:** National Engineering Research Center of Immunological Products, Department of Microbiology and Bio-Chemical Pharmacy, College of Pharmacy, Army Medical University, Chongqing 400038, China

**Keywords:** *Klebsiella pneumoniae*, FimA antigen, B-cell epitope, fine mapping, protective analysis

## Abstract

**Background/Objectives:** *Klebsiella pneumoniae* (*K. pneumoniae*) is a leading cause of serious hospital-acquired and community-acquired infections, with limited treatment options, especially for immunocompromised and critically ill patients. No licensed vaccine is currently available. The FimA antigen, a key fimbrial subunit essential for bacterial adhesion and invasion, represents a promising vaccine target. However, little is known about the immunodominant antibody responses against invasive *K. pneumoniae.* This study aimed to evaluate the immunogenicity and protective efficacy of recombinant FimA protein, to fine-map its immunodominant linear B-cell epitopes, and to assess the individual and combined protective capacity of these epitopes against both standard and clinically isolated *K. pneumoniae* strains. **Methods:** A murine model of lethal *K. pneumoniae* challenge was used. Recombinant FimA protein was administered to evaluate immunogenicity and protective efficacy. Immunodominant linear B-cell epitopes were identified by overlapping peptide ELISA using immune antisera. The identified epitopes were synthesized and conjugated to keyhole limpet hemocyanin (KLH). Mice were immunized with individual epitope-KLH conjugates or a mixture of all four, then challenged with the standard strain ATCC700721 or with multiple clinical isolates of distinct multilocus sequence types (MLST). Epitope-specific antibody responses (total IgG and IgG subclasses) and survival rates were measured. **Results:** Immunization with full-length recombinant FimA conferred 90% protection against lethal challenge with the standard strain ATCC700721 and induced robust IgG1-dominant antibody responses. Four novel immunodominant linear B-cell epitopes were identified: FimA_97–114_, FimA_103–120_, FimA_109–126_, and FimA_145–160_. Structural mapping revealed that the first three epitopes reside within the α-helical region, while FimA_145–160_ is located in the β-sheet domain. These epitopes are highly conserved, exhibiting 100% sequence identity across 36 diverse *K. pneumoniae* strains. Among individual epitope-KLH conjugates, FimA_109–126_-KLH induced the highest epitope-specific antibody titers, followed by FimA_103–120_-KLH. Immunization with a mixture of all four epitope-KLH conjugates elicited significant cross-protection against multiple clinical isolates, achieving survival rates of 60%, 50%, 50%, and 40% against strains 10CYZ, 13LGY, 19ZXQ, and 22CZY, respectively. Protective immunity was primarily associated with IgG1 subtype responses. **Conclusions:** This study provides the first fine-mapping and protective evaluation of immunodominant linear B-cell epitopes within *K. pneumoniae* FimA. The identification of highly conserved, functionally relevant B-cell epitopes and the demonstration of cross-protection conferred by a multi-epitope formulation underscore the potential of FimA-based epitope-driven vaccines. These findings offer a promising strategy for the development of broadly protective vaccines against *K. pneumoniae* infections.

## 1. Introduction

*Klebsiella pneumoniae* (*K. pneumoniae*), a Gram-negative bacterium, is a leading cause of hospital-acquired and community-acquired infections [1]. *K. pneumoniae* causes a wide range of infections, including pneumonias, urinary tract infections, bacteremias, and liver abscesses [2]. Hypervirulent *K. pneumoniae* (hvKp) is an evolving pathotype that is more virulent than classical *K. pneumoniae* (cKp), which usually infects individuals from the community who are often healthy [3]. Currently, antibiotics such as meropenem remain the first-line treatment for *K. pneumoniae* infections [4]. Globally, over 8% of individuals with *K. pneumoniae* bloodstream infections exhibit resistance to carbapenem antimicrobials. The emergence of multidrug-resistant (MDR) and carbapenem-resistant strains has limited treatment options, making infections difficult to control [5]. However, given the deteriorating situation of antimicrobial resistance, exploring novel therapeutic strategies is particularly necessary and prudent. Vaccines targeted against *K. pneumoniae* are considered to be a priority by the World Health Organization [6]. However, no licensed vaccine is currently available.

To date, a variety of vaccines, including killed or whole-cell mutant, conjugate/polysaccharide, outer membrane proteins, fimbriae, and so on, have been studied for *K. pneumoniae* prevention in animals and/or humans in some clinical trials [7,8]. Among these proteins, FimA, which is a type I fimbriae protein, could be a promising vaccine candidate [9]. Type I fimbriae are composed of heteropolymeric adhesion organelles that include FimH, a mannose-specific lectin, but are composed mainly of FimA [10].

Advances in antigen discovery have facilitated the identification of potential vaccine candidates for Klebsiella infections. Recent studies have focused on characterizing immunodominant epitopes and evaluating their protective efficacy, laying the groundwork for the development of next-generation vaccines [11]. Subsequent studies revealed that these epitopes elicited a strong antibody response in mice, demonstrated significant protective efficacy in infection models, and exhibited cross-reactivity with sera from naturally infected patients.

In this study, we screened and mapped the B-cell linear epitopes of FimA, the principal protein of type I fimbriae in *K. pneumoniae*. Further research efforts are needed to address the challenges associated with Klebsiella infections, including vaccine optimization, clinical trials, and implementation strategies. Collaborative initiatives between researchers, clinicians, and policymakers are essential to mitigate the global impact of Klebsiella infections and safeguard public health.

## 2. Materials and Methods

### 2.1. Ethics Statements

All animal and human experiments were approved by the Laboratory Animal Welfare and Ethics Committee of the Army Medical University (Chongqing, China; Permit AMUWEC20230478). The study was conducted in accordance with the local legislation and institutional requirements.

### 2.2. Animals

For this study, 6–8-week-old female BALB/c mice were purchased from Hunan SJA Laboratory Animal Co., Ltd. (Changsha, China). For euthanasia, mice were anesthetized with isoflurane and then killed with CO_2_.

### 2.3. Expression and Purification of FimA Protein

The FimA antigen was expressed and purified as follows. The recombinant full-length FimA protein was prepared using genetic recombination techniques, following a comprehensive analysis of the gene sequences of *K. pneumoniae* strains through bioinformatics methods. Gene and amino acid information for the FimA antigen was obtained from NCBI (Sequence ID: WP_032434944.1). Specific primers were designed to clone the target gene of FimA, which was digested with BamHI and XhoI and subsequently ligated into the pGEX-6P-1 plasmid. Escherichia coli was used as the host cell to construct the recombinant strain. Target protein expression was induced with a GST tag, followed by enzymatic cleavage to remove the GST tag. The recombinant FimA protein lacking the GST tag had a molecular weight of 17.3 kDa. The purified protein was further analyzed via sodium dodecyl sulfate-polyacrylamide gel electrophoresis (SDS-PAGE) to confirm its molecular weight, and a FimA protein with >95% purity was successfully prepared. The concentration of FimA protein was measured using the BCA method.

### 2.4. Peptide Synthesis

Eighteen-mer peptides with 12 amino acid length overlaps to cover the full lengths of FimA (NCBI Sequence ID: WP_032434944.1) were synthesized and purified by GL Biochem Ltd. (Shanghai, China). OVA_192–201_ (EDTQAMPFRV) peptide was used as a negative control. The purity of these peptides was expected to be 95% or higher. The peptides were dissolved in dimethyl sulfoxide at a concentration of 0.5 mg/mL and stored at −80 °C before use.

### 2.5. Establishment of a K. pneumoniae Infection Model

BALB/c mice of the same batch were subjected to multiple doses of infection, with n = 10. Single colonies were obtained using the streak plate method, and picked colonies were cultured overnight at 37 °C, 150 rpm. The next day, secondary activation was performed with culture at 37 °C, 220 rpm for 4.5 h, followed by centrifugation at 8000 rpm for 5 min to remove the supernatant. The pellet was resuspended in PBS, centrifuged again, and the supernatant was removed. The bacterial suspension was resuspended in 1 mL PBS and adjusted to an OD value between 0.95~1.05. Multiple doses were set for screening lethal and sublethal doses.

### 2.6. Immunization

Twenty female BALB/c mice, aged 6 to 8 weeks and weighing between 18 and 20 g (purchased from Weitonglihua, Beijing, China), were selected and randomly assigned to two groups. The vaccine was formulated by combining 75 μg of FimA protein with 0.5 mg of Al(OH)_3_ adjuvant, followed by adsorption at room temperature on a shaker for 2 h. Each mouse in the immunization group received an intramuscular injection of 200 μL of the vaccine (100 μL into each hind leg), while the control group was administered the same volume of PBS via the same route and regimen. The immunization schedule consisted of three doses administered on days 0, 7, and 14. Blood samples were collected from the ocular region on the 7th day post-immunization. The ocular blood was then centrifuged at 4 °C and 6000 rpm for 5 min to obtain the serum.

### 2.7. Challenge Study

The standard *K. pneumoniae* strain (ATCC700721, TaxID: 272620) was purchased from the American Type Culture Collection. One week after the final booster immunization, mice were challenged with lethal doses of ATCC700721. Survival rates were observed for 7 days in the FimA and PBS groups (n = 10). Sublethal doses were administered, and lung tissues were collected 48 h later. Organ homogenates were prepared in 1 mL PBS, serially diluted 5-fold in a 96-well plate (NEST), and plated in pre-inverted agar plates. Bacterial colony-forming ability was observed after incubation at 37 °C for 16 h (n = 8).

### 2.8. Detection of Specific Antibodies and Their Subtypes Induced by Recombinant FimA Protein Immunization Using Indirect ELISA

One week after the final immunization, blood samples were collected to obtain serum samples. An enzyme-linked immunosorbent assay (ELISA) was conducted to determine the potency of FimA-specific immunoglobulin G (IgG). In brief, antigen FimA was adjusted to 5 μg/mL in 50 mM carbonate buffer (pH 9.6), and 100 μL per well was added to coat a 96-well microplate (Corning, New York, NY, USA) overnight at 4 °C. Non-specific binding was blocked by incubating with 2% bovine serum albumin (BSA; m/v) at 37 °C for 2 h.

Serum samples were diluted in phosphate-buffered saline with Tween 20 (PBST) in a two-fold serial dilution manner, starting from 1:1000, resulting in eight dilution gradients, which were used as the primary antibodies. HRP-conjugated goat anti-mouse IgG (Bioss, Beijing, China) was used as the secondary antibody at a 1:10,000 dilution. Absorbance was measured at 450 nm (OD450 nm), and titers were determined as the highest dilution that produced an absorbance value exceeding 2.1 times that of the pre-immune serum.

To analyze the subtypes of FimA-specific antibodies, serum samples diluted at 1:1000 were used as the primary antibodies, and HRP-conjugated goat anti-mouse IgG1, IgG3, IgG2a, IgG2b, IgM, and IgA antibodies (Alpha Diagnostic, San Antonio, TX, USA) were used as the secondary antibodies.

### 2.9. Bacterial Burden

Seven days after the last immunization, mice in each group were infected with 1 × 10^6^ CFU of ATCC700721 (n = 8), and lungs were harvested 48 h post infection. The bacterial burden in the organs was quantified by preparing organ homogenates in PBS and plating 5-fold serial dilutions on LB plates. The colonies were counted after 24 h of incubation at 37 °C. The number of CFU per tissue (CFU/organ) was calculated from each plate.

### 2.10. Fine-Mapping of B-Cell Immune-Dominant Epitopes of FimA

The concentration of overlapping peptides for coating was adjusted to 5 μg per well with the peptide pool as a positive control. After coating the plate, washing, blocking, and subsequent washing, the obtained antisera from the FimA immunized mice (n = 15) were diluted to 1:500 and then incubated for 1 h. Following washing, HRP-conjugated goat anti-mouse IgG (purchased from Bioss, catalog number bs-0296G-HRP) at a dilution of 1:10,000 was added, followed by washing and the addition of TMB substrate chromogenic solution (Beyotime, Shanghai, China/Bioss, Beijing, China). After terminating the reaction, OD values were read at 450 nm. A positive overlapping peptide was defined as (OD value of 18 amino acid overlapping peptide − OD value of blank control)/(OD value of negative peptide − OD value of blank control) ≥ 2.1.

### 2.11. Structural Localization and Sequence Alignment of the Immunodominant Epitopes

The crystal structure of FimA (PDB code: 6JZK) was obtained from the protein data bank (PDB). Immunodominant epitopes were located on these structures using the PyMOL 1.1 program.

Amino acid sequences of the FimA protein from 30 strains of *K. pneumoniae* were retrieved from the GenBank database. The NCBI’s Basic Local Alignment Search Tool (BLAST) was used for amino acid sequence alignment analysis. The website address is https://blast.ncbi.nlm.nih.gov/Blast.cgi, accessed on 7 March 2026.

### 2.12. Protective Analysis of Keyhole Limpet Hemocyanin (KLH)-Conjugated Epitope Peptides

Keyhole limpet hemocyanin (KLH) is often used as a carrier protein to enhance the immunogenicity of epitope peptides. In this study, we investigated the immunogenicity and protective efficacy of KLH-conjugated epitope peptides in a preclinical model. Epitope peptides were conjugated to KLH and used to immunize mice. Serum samples were collected after immunization, and the presence of peptide-specific antibodies was determined using enzyme-linked immunosorbent assay (ELISA). The protective efficacy of the immunization was evaluated by challenging the immunized mice with the corresponding pathogen and assessing survival rates, bacterial load, and histopathological changes.

KLH-conjugated epitope peptides were adjusted to a dose of 100 μg per mouse for immunization. The adjuvant used was Al(OH)_3_ (InvivoGen, San Diego, CA, USA), administered at a dose of 50 μL. Immunization was administered via intramuscular injection, with three immunizations given at days 0, 7, and 14. One week after the final booster immunization, mice were challenged with a lethal dose of the ATCC700721 of the standard strain. This was done to observe the 7-day survival rates of four types of KLH-conjugated epitope peptides, mixed peptides, and the PBS + Al(OH)_3_ group (n = 10). Sublethal dose challenges were also conducted. Sublethal dose challenges were followed by histopathological examination (n = 8), following the methods outlined on the first page. The mixed peptides group includes all of the above immunodominant B-cell epitopes of FimA.

### 2.13. Indirect ELISA Method for Detecting Specific IgG Antibody Subtypes Against Dominant Epitope Peptide-KLH Conjugated Protein

One week after the final immunization, blood samples were collected to obtain serum samples. An enzyme-linked immunosorbent assay (ELISA) was performed to measure the potency of FimA-specific immunoglobulin G (IgG). In brief, the dominant epitope peptide-KLH was adjusted to a concentration of 5 μg/mL in 50 mM carbonate buffer (pH 9.6) and 100 μL of the solution was added to each well of a 96-well microplate (Corning, USA), which was then incubated overnight at 4 °C. Non-specific binding was blocked by incubating with 2% bovine serum albumin (BSA; m/v) at 37 °C for 2 h. Serially dilute serum samples from the immunized mice in PBST, starting from 1:1000, to obtain eight dilution gradients. Add 100 μL of each diluted serum sample to the coated wells. Incubate the microplate at 37 °C for 1 h to allow binding of specific IgG antibodies to the immobilized peptide-KLH conjugated protein. Wash the wells three times with PBST to remove unbound antibodies.

Add horseradish peroxidase (HRP)-conjugated goat anti-mouse IgG1, IgG2a, IgG2b, IgG3, IgM, and IgA antibodies (Alpha Diagnostic) at a dilution of 1:10,000 to the wells and incubate at 37 °C for 1 h. Wash the wells three times with PBST to remove unbound secondary antibodies. Add the substrate solution (e.g., TMB substrate) and incubate in the dark for 15–30 min at room temperature. Add a stop solution (e.g., 2 M sulfuric acid) to terminate the reaction.

### 2.14. Analysis of Inflammatory Cytokine Levels in Immune Peptide-KLH Conjugate Immunized Mice After Infection Using LEGENDplex™

One week after the final immunization of the immune peptide-KLH conjugate, mice were infected with sublethal doses of ATCC700721. Two days post-infection, each mouse was euthanized with carbon dioxide, and bronchoalveolar lavage fluid (BALF) was collected, with each collection totaling 500 μL, and was performed three times. Using the LEGENDplex™ panel from Biolegend, pro-inflammatory cytokine levels of BALF, including IFN-γ, TNF-α, IL-2, IL-4, IL-5, IL-6, IL-10, IL-13, IL-17A, IL-17F, and IL-22, were determined (n = 5). Prior to testing, samples were allowed to thaw only once at room temperature. All assays were performed in duplicate, and the testing methodology utilized One AVAOA, with results presented as mean ± SEM.

### 2.15. Broad-Spectrum Immunoprotective Analysis of Mixed Immune Dominant Peptides Against Clinical Strains of K. pneumoniae

The mixed peptides include FimA_97–114_, FimA_103–120_, FimA_109–126_, and FimA_145–160_. Analyzing the broad-spectrum protective efficacy of mixed peptides conjugated with KLH (Mix-Peptides) against different clinical strains of *K. pneumoniae* compared to the corresponding control groups.

### 2.16. Analysis of Immune-Dominant Peptides of FimA Antigen in Human Convalescent Sera of K. pneumoniae

Serum samples were collected from 15 patients with *Klebsiella pneumoniae* infection to analyze the titers and subtypes of FimA-specific antibodies, and the strongest responsive immune-dominant peptides were analyzed using the indirect ELISA method with overlapping peptides. And statistical analysis was performed using one-way ANOVA with Tukey’s HSD post hoc correction after confirming normality (Shapiro–Wilk test).

### 2.17. Statistical Analysis

Statistical analyses were performed using GraphPad Prism 8.0. All data are presented as the means ± standard deviation (S.D.). Data were analyzed using a *t*-test and one-way ANOVA with Bonferroni correction. *p* < 0.05 was considered statistically significant.

## 3. Results

### 3.1. Analysis of Survival Rate in BALB/c Mice Infected with ATCC700721 at Different Infection Concentrations

In order to evaluate the survival rate in BALB/c mice infected with ATCC700721, BALB/c mice were immunized intramuscularly on days 0, 7, and 14 with recombinant FimA formulated with Al(OH)_3_ adjuvant. One week after the final immunization, the mice were challenged with a lethal dose of the ATCC700721 via tracheal intubation.

**Figure 1:**
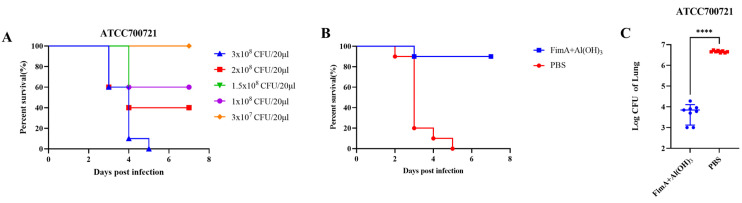
Analysis of survival rate in BALB/c mice infected with ATCC700721 at different infection concentrations. BALB/c mice were immunized intramuscularly on days 0, 7, and 14 with recombinant FimA formulated with Al(OH)_3_ adjuvants. One week after the final immunization, the mice were challenged with a lethal dose of ATCC700721 via tracheal intubation. (**A**) Naive mice challenged with five different concentrations of the ATCC700721 standard strain showed varying survival rates. (**B**) Seven days after the third immunization, mice challenged with the lethal dose of the ATCC700721 showed a 90% protection rate with the FimA protein. (**C**) Bacterial clearance in the immunized group with FimA plus Al(OH)_3_ compared to the PBS Al(OH)_3_ group post-infection with the sublethal dose of the ATCC700721 standard strain (****, *p* < 0.0001). Mice immunized with FimA+Al(OH)_3_ adjuvant showed significantly lower bacterial loads in their lungs after bronchial intubation with the sublethal dose of the ATCC700721 strain compared to the PBS control group.

As shown in Figure 1A, mice challenged with five different concentrations of the ATCC700721 standard strain showed varying survival rates. Based on these results, the concentration of 3 × 10^8^ CFU/20 μL was selected as the lethal challenge dose for subsequent survival experiments, as it resulted in 100% mortality within five days. Conversely, 3 × 10^7^ CFU/20 μL, which caused no mortality, was chosen as the sublethal dose for evaluating bacterial burden, histopathology, and cytokine responses. All mice challenged with 3 × 10^8^ CFU/20 μL died by the fifth day, while survival rates for doses of 2 × 10^8^ CFU/20 μL and 1.5 × 10^8^ CFU/20 μL were 40%, and 1 × 10^8^ CFU/20 μL showed a survival rate of 60%. The lowest dose tested, 3 × 10^7^ CFU/20 μL, resulted in 100% survival. Therefore, 3 × 10^8^ CFU/20 μL was chosen as the lethal dose of the ATCC700721 standard strain for subsequent experiments to observe the survival rate of immunized mice seven days after the final immunization. A sublethal dose of 3 × 10^7^ CFU/20 μL was selected for observing bacterial load, pathological damage, and cytokine detection.

Seven days after the third immunization, mice challenged with the lethal dose of the ATCC700721 showed a 90% protection rate with the FimA protein (Figure 1B). The immunized group with FimA showed significantly better bacterial clearance compared to the PBS group when challenged with the sublethal dose of the ATCC700721 standard strain (*p* < 0.0001) (Figure 1C). Mice immunized with FimA + Al(OH)_3_ adjuvant showed significantly lower bacterial loads in their lungs after tracheal intubation intubation with the sublethal dose of the ATCC700721 strain compared to the PBS control group (Figure 1C). In summary, these results indicate that immunization with FimA along with Al(OH)_3_ adjuvant provides stronger protection against attacks by the ATCC700721 strain compared to using PBS alone as a control.

### 3.2. Analysis of Specific Antibody and Subtype Levels Induced by Recombinant FimA Protein Immunization

To characterize the humoral immune responses induced by FimA formulated with Al(OH)_3_ adjuvant, serum samples were collected from immunized BALB/c mice seven days after the final immunization. ELISA was performed to measure the potency of FimA-specific IgG and its subtypes.

**Figure 2:**
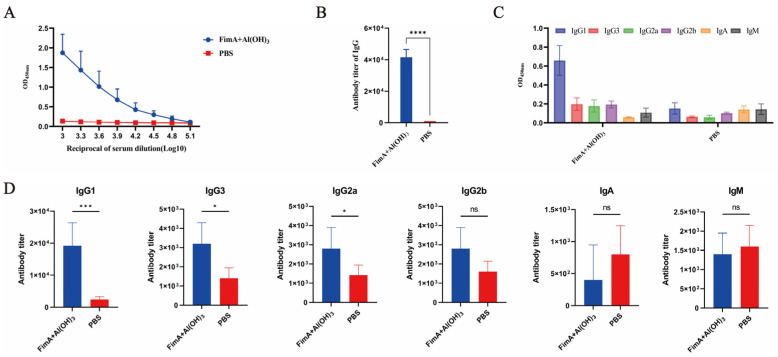
Analysis of specific antibody and subtype levels induced by recombinant FimA protein immunization. Serum samples were collected from immunized BALB/c mice seven days after the final immunization. An enzyme-linked immunosorbent assay (ELISA) was performed to measure the potency of FimA-specific immunoglobulin G (IgG) and its subtypes. (**A**) Reciprocal of serum dilution in all immunized mice. (**B**) Antibody titer of IgG in all immunized mice. (**C**) The OD450 of each subtype of antibodies in the serum of mice immunized with FimA plus Al(OH)_3_. (**D**) The titers of each subtype of antibodies in the serum of mice immunized with FimA plus Al(OH)_3_ (*, *p* < 0.05; ***, *p* < 0.001; ****, *p* < 0.001; ns, not significant).

The OD450 absorbance detected at various serial dilutions showed a progressive decrease as the dilution factor increased (Figure 2A). As depicted in Figure 2B, all mice immunized with FimA plus Al(OH)_3_ exhibited elevated levels of specific antibodies, and the average titer of antibodies in mice immunized with FimA plus Al(OH)_3_ was 41,600. The titers of FimA-specific antibodies in mice from the FimA + Al(OH)_3_ group were significantly higher than those in the PBS + Al(OH)_3_ group (*p* < 0.0001). The levels of each subtype of antibodies in the serum of mice immunized with FimA plus Al(OH)_3_ showed a similar trend to that of total antibodies, with IgG1 being the predominant subtype of the antibodies (Figure 2C,D).

### 3.3. Identification of B-Cell Immunodominant Epitopes of FimA

To elucidate adjuvant-driven differences in FimA-specific immunity, we mapped immunodominant linear B-cell epitopes using serum samples collected on day 7 after the final immunization from BALB/c mice immunized with FimA formulated with Al(OH)_3_. We would like to clarify that the overlapping peptide ELISA approach is a well-established and widely accepted method for mapping immunodominant B-cell epitopes. It has been successfully employed in numerous published studies.

**Figure 3:**
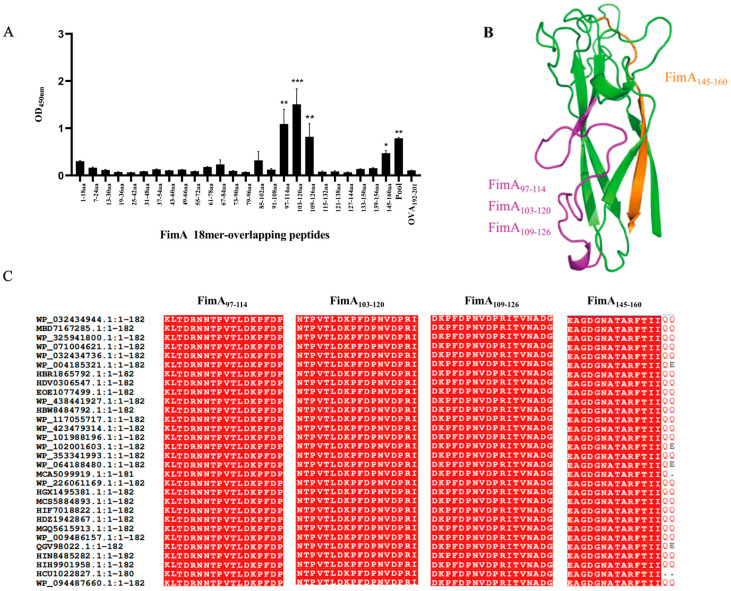
Identification of B-cell immunodominant epitopes of FimA. To elucidate adjuvant-driven differences in FimA-specific immunity, we mapped immunodominant linear B-cell epitopes using antisera collected on day 7 after the final immunization from BALB/c mice immunized with FimA formulated with Al(OH)_3_. (**A**) Fine mapping of B-cell immunodominant epitopes of FimA. There were four positive peptides: FimA_97–114_, FimA_103–120_, FimA_109–126_, and FimA_145–160_, which showed significantly higher readings compared to other dominant peptides, indicating their dominance as epitopes. OVA_192–201_ serves as a negative irrelevant peptide. (**B**) Localization of B-cell immunodominant epitopes of FimA in its three-dimensional structure. To explore the structural basis for the observed differences in immunodominance hierarchy and their correlation with protective efficacy, we mapped the four epitopes onto the crystal structure of FimA (PDB ID: 6JZK) using PyMOL1.1 software. The immunedominant peptides FimA_97–114_, FimA_103–120_, FimA_109–126_, and FimA_145–160_ are located on the outer surface of the FimA three-dimensional crystal structure. Among them, FimA_97–114_, FimA_103–120,_ and FimA_109–126_ were on the α-helical region of FimA, while FimA_145–160_ was on the β-sheet structure of FimA. (**C**) Amino acid sequence conservation analysis of B-cell immunodominant epitopes of FimA. 30 randomly selected *K. pneumoniae* strains were obtained from the GenBank database. The regions shown in red represent amino acid sequence identity, and the amino acid sequences of these immunodominant peptides are highly conserved among 30 *K. pneumoniae* strains, with a sequence identity reaching 100%. (*, *p* < 0.05; **, *p* < 0.01; ***, *p* < 0.001).

As shown in Figure 3A, four positive peptides, FimA_97–114_ (KLTDRNNTPVTLDKPFDP), FimA_103–120_ (NTPVTLDKPFDPNVDPRI), FimA_109–126_ (DKPFDPNVDPRITVNADG), and FimA_145–160_ (EAGDGNATARFTIIQQ), showed significantly higher readings compared to other dominant peptides, indicating their dominance as epitopes. In the figure, OVA_192–201_ serves as a negative irrelevant peptide.

### 3.4. Localization of B-Cell Immunodominant Epitopes of FimA in Its Three-Dimensional Structure

To explore the structural basis for the observed differences in immunodominance hierarchy and their correlation with protective efficacy, we mapped the Four epitopes onto the crystal structure of FimA (PDB ID: 6JZK) using PyMOL software. As shown in Figure 3B, the immune-dominant peptides FimA_97–114_, FimA_103–120_, FimA_109–126_, and FimA_145–160_ are located on the outer surface of the FimA three-dimensional crystal structure. Among them, FimA_97–114_, FimA_103–120,_ and FimA_109–126_ were on the α-helical region of FimA, while FimA_145–160_ was on the β-sheet structure of FimA.

### 3.5. Amino Acid Sequence Blast Analysis of B-Cell Immunodominant Epitopes of FimA

To evaluate the conservation of the identified immunodominant epitopes, we performed a sequence alignment of the amino acid sequences of FimA from 30 randomly selected *K. pneumoniae strains* obtained from the GenBank database.

As shown in Figure 3C, the amino acid sequences of these immunodominant peptides are highly conserved among 30 *K. pneumoniae* strains, with a sequence identity reaching 100%, suggesting their potential to provide broad-spectrum protection.

### 3.6. Analysis of Immunoprotective Capability of Dominant Epitope Peptides-KLH Protein

**Figure 4:**
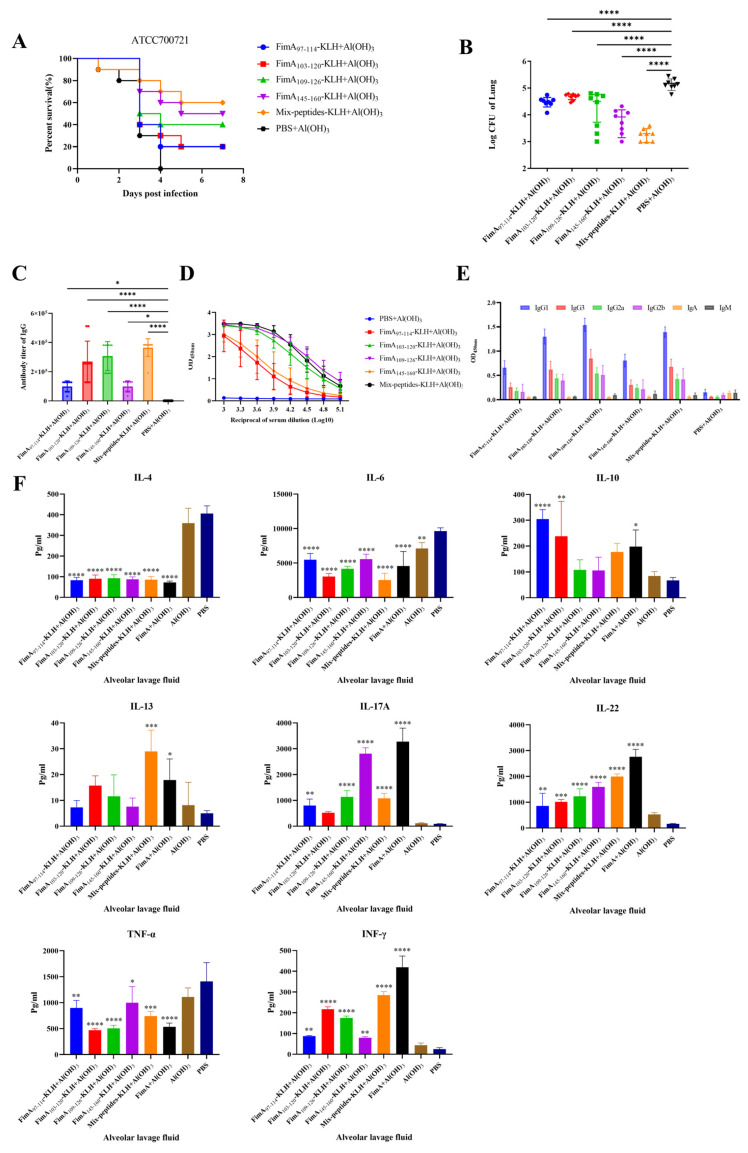
Analysis of immunoprotective capability of immunodominant epitope peptides-KLH protein. To analyze the immunoprotective properties of immunodominant epitope peptides, the peptides were conjugated with the KLH protein. The mix-peptides-KLH includes FimA_97–114_-KLH, FimA_103–120_-KLH, FimA_109–126_-KLH and FimA_145–160_-KLH. Subsequently, BALB/c mice were immunized with the conjugated protein to assess the survival rate of actively immunized mice following ATCC700721 infection. (**A**) Survival rate of the immunized mice after ATCC700721 infection. Compared to the PBS + Al(OH)_3_ group, the statistical analysis revealed the following differences in these epitope-KLH immunized groups: FimA_109–126_-KLH + Al(OH)_3_ (****, *p* < 0.0001)**,** FimA_145–160_-KLH + Al(OH)_3_ (****, *p* < 0.0001), and Mix-Peptides + Al(OH)_3_ (****, *p* < 0.0001). (**B**) The amount of the *K. pneumoniae* in the immunized mice after ATCC700721 infection. Compared to the PBS + Al(OH)_3_ group, the statistical analysis revealed the following differences in clearing the bacteria in the infected lung: FimA_109–126_-KLH + Al(OH)_3_ (****, *p* < 0.0001)**,** FimA_145–160_-KLH + Al(OH)_3_ (****, *p* < 0.0001), and Mix-Peptides + Al(OH)_3_ (****, *p* < 0.0001). (**C**) The average levels of epitope-specific antibodies in the mice immunized with the single peptide-KLH or mixed peptides-KLH. (**D**) Reciprocal of serum dilution in the mice immunized with the single peptide-KLH or mixed peptides-KLH immunized mice. (**E**) The levels of each subtype of antibodies in the antisera of mice immunized with the single peptide-KLH or mixed peptides-KLH immunized mice. (**F**) Cytokine profiling analysis of the antisera in the mice immunized with the single peptide-KLH or mixed peptides-KLH immunized mice. One week after the final immunization, mice were challenged with a sublethal dose of ATCC700721, and the levels of proinflammatory cytokines in BALF were measured 48 h post-infection. Statistical significance was annotated using GraphPad Prism 8.0, and significance levels are indicated by asterisks above the bars in the bar graph. (*, *p* < 0.05; **, *p* < 0.01; ***, *p* < 0.001, ****, *p* < 0.0001).

To analyze the immunoprotective properties of dominant epitope peptides, the peptides were conjugated with KLH protein. The Mix-Peptides-KLH includes FimA_97–114_-KLH, FimA_103–120_-KLH, FimA_109–126_-KLH and FimA_145–160_-KLH. Subsequently, BALB/c mice were immunized with the conjugated protein to assess the survival rate of actively immunized mice following ATCC700721 infection. As shown in Figure 4A, in the group actively immunized with Mix-Peptides-KLH plus Al(OH)_3_ adjuvant, the survival rate was 60%, while those immunized with FimA_145–160_-KLH combined with Al(OH)_3_ adjuvant showed a survival rate of 50%, FimA_109–126_-KLH combined with Al(OH)_3_ adjuvant showed a survival rate of 40%, and 20% for those immunized with FimA_97–114_-KLH or FimA_103–120_-KLH or PBS combined with Al(OH)_3_ adjuvant. Compared to the PBS + Al(OH)_3_ group, the statistical analysis revealed the following differences in these epitope-KLH immunized groups: FimA_109–126_-KLH + Al(OH)_3_ (*p* < 0.0001)**,** FimA_145–160_-KLH + Al(OH)_3_ (*p* < 0.0001), and Mix-Peptides + Al(OH)_3_ (*p* < 0.0001).

To assess the bacterial clearance ability induced by vaccination, mice immunized with epitopes-KLH plus adjuvant Al(OH)_3_ were challenged with a sublethal dose of ATCC700721, and lung bacterial loads were quantified. As expected, all adjuvanted FimA epitope-KLH + Al(OH)_3_ groups showed significantly lower pulmonary bacterial burdens than the PBS + Al(OH)_3_ control (Figure 4B). Compared to the PBS + Al(OH)_3_ group, the statistical analysis revealed the following differences in clearing the bacteria in the infected lung: FimA_109–126_-KLH + Al(OH)_3_ (*p* < 0.0001)**,** FimA_145–160_-KLH + Al(OH)_3_ (*p* < 0.0001), and Mix-Peptides-KLH + Al(OH)_3_ (*p* < 0.0001).

As shown in Figure 4C,D, under the same Al(OH)_3_ adjuvant-assisted immunization, the average levels of epitope-specific antibodies were determined as follows: Anti-FimA_97–114_: 1:99,200, Anti-FimA_103–120_: 1:268,800, Anti-FimA_109–126_: 1:307,200, Anti-FimA_145–160_: 1:99,200, Anti-Mix-Peptides: 1:364,800. These results demonstrate variations in the potency levels of epitope-specific antibodies elicited by different epitope-KLH conjugates. Specifically, FimA_109–126_-KLH exhibited the highest level of the epitope-specific antisera, followed by FimA_103–120_-KLH and Mix-Peptides-KLH. FimA_97–114_-KLH and FimA_145–160_-KLH showed relatively lower levels of epitope-specific antibodies. In antisera of the mice immunized with every single epitope-KLH plus Al(OH)_3_, IgG1 was the dominant subtype of the IgG antibodies.

One week after the final immunization, mice were challenged with a sublethal dose of ATCC700721, and the levels of proinflammatory cytokines in bronchoalveolar lavage fluid (BALF) were measured 48 h post-infection. As shown in Figure 4E, cytokine profiling analysis indicated that in immunized mice infected with ATCC700721, IL-13 exhibited an increasing trend, with a significant difference noted in the Mix-Peptides group. In contrast, levels of IL-4 and IL-6 were significantly decreased. IL-10 demonstrated an upward trend, with significant differences observed in the FimA_103–120_ and FimA_109–126_ groups. Notably, IL-13 was significantly elevated, particularly in the Mix-Peptides group. Meanwhile, IFN-γ, IL-17, and IL-22 were substantially upregulated. These alterations in cytokine profiles provide critical insights into how the vaccine influences post-infection immune protective mechanisms. Immunization with a vaccine composed of dominant epitope peptides conjugated to KLH protein facilitated the rapid activation of Th1- and Th17-type immune responses in infected mice, as evidenced by the significant elevation of IFN-γ, IL-17, and IL-22, alongside the suppression of IL-4. The increase in IL-13 may be linked to atypical functional activation of the Th2-type response or non-Th2 sources. Furthermore, the upregulation of IL-13, along with the increasing trend of IL-10 and the reduction in IL-6, collectively reflects the body’s negative feedback regulation of the inflammatory response, as well as the processes involved in inflammation resolution and mucosal protection. Following infection, immunized mice exhibited a significant elevation of IL-13, particularly in the Mix-Peptides group, alongside a marked reduction in IL-4. Although IL-4 and IL-13 are typically co-expressed by activated Th2 cells and share downstream signaling through the IL-4 receptor α chain (IL-4Rα) [12,13,14], growing evidence indicates that IL-13 can also be produced by non-Th2 cell populations, including group 2 innate lymphoid cells (ILC2s), mast cells, basophils, and injured epithelial cells [15,16]. In the context of bacterial infection and vaccination, suppression of Th2-polarizing signals (as reflected by low IL-4) does not necessarily preclude IL-13 production from these alternative sources. The elevated IL-13 observed here, together with an increasing trend of IL-10 and a significant decrease in IL-6, suggests a role in tissue repair, epithelial barrier maintenance, and negative regulation of excessive inflammation [17,18]. Importantly, protective Th1 and Th17 responses (indicated by elevated IFN-γ, IL-17, and IL-22) remained intact, demonstrating that the vaccine did not induce a non-protective Th2 bias. Instead, the selective increase in IL-13 likely contributes to mucosal healing and resolution of inflammation without compromising anti-bacterial immunity [18,19]. Collectively, the dissociation of IL-13 from IL-4 in this study reflects a functionally favorable immune modulation rather than an aberrant Th2 response.

### 3.7. Broad-Spectrum Immunoprotective Analysis of Mixed Immunodominant Peptides-KLH Against Clinical Strains of K. pneumoniae 

To assess the broad-spectrum immunoprotection of the mixed immunodominant peptides-KLH against clinical strains of *K. pneumoniae*, various clinical isolates were chosen based on MLST. One week after the final immunization, the mice were challenged with a lethal dose of the clinical isolates via tracheal intubation.

**Figure 5:**
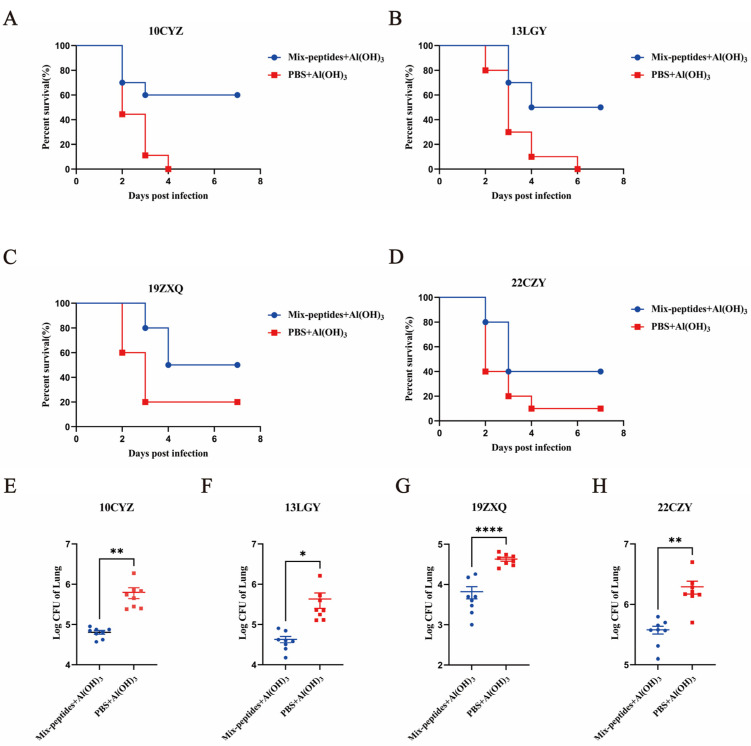
Broad-spectrum immunoprotective analysis of mixed immunodominant peptides-KLH against clinical strains of *K. pneumoniae*. To assess the broad-spectrum immunoprotection of the mixed immunodominant peptides-KLH against clinical strains of *K. pneumoniae*, various clinical isolates were chosen based on MLST. One week after the final immunization, the mice were challenged with a lethal dose of the clinical isolates via tracheal intubation. Survival rate of the immunized mice after clinical isolate infections. Comparing with the corresponding control groups, Mix+Peptides-KLH showed a survival rate of 60% against clinical isolate 10CYZ (**A**), 50% against 13LGY (**B**), 50% against 19ZXQ (**C**), 40% against 22CZY (**D**). Compared with the PBS + Al(OH)_3_ group, the statistical results of bacterial burden in the lungs of mice immunized with the mixed peptide + Al(OH)_3_ following challenge with different strains are shown below: 10CYZ challenge group (**, *p* =0.0099), 13LGY (*, *p* = 0.0471), 19ZXQ (****, *p* < 0.0001), 22CYZ (**, *p* = 0.0051) (**E**–**H**).

The mixed peptides-KLH include FimA_97–114_-KLH, FimA_103–120_-KLH, FimA_109–126_-KLH, and FimA_145–160_-KLH. Comparing with the corresponding control groups, Mix+Peptides-KLH showed a survival rate of 60% against clinical isolate 10CYZ (Figure 5A), 50% against 13LGY (Figure 5B), 50% against 19ZXQ (Figure 5D), 40% against 22CZY (Figure 5C), and 60% against ATCC700721(Figure 4A), respectively. Compared with the PBS + Al(OH)_3_ group, statistical analysis of the survival rates of mice immunized with Mix-Peptides + Al(OH)_3_ after different strain challenges were as follows: 10CYZ challenge group (*p* = 0.0099), 13LGY (*p* = 0.0471), 19ZXQ (*p* < 0.0001), 22CYZ (*p* = 0.0051), ATCC700721 (*p* = 0.0292). In addition, analysis of IgG Antibody Subtypes in Mixed Immune-Dominant Peptide-KLH Immunized Mice revealed that the IgG1 subtype predominated.

### 3.8. Analysis of Immunodominant Peptides of FimA Antigen in Clinical Human Convalescent Sera of K. Pneumoniae After Infection

**Figure 6:**
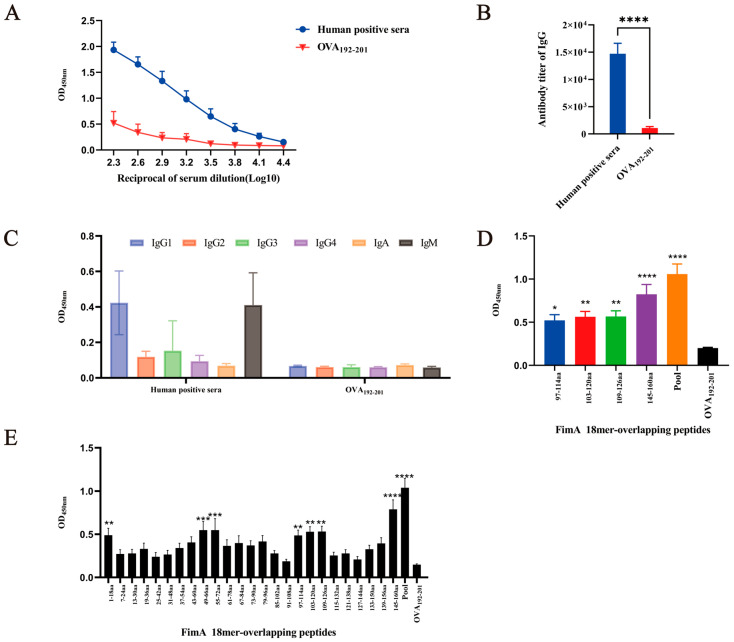
Analysis of immunodominant peptides of FimA antigen in clinical human convalescent sera after *K. pneumoniae* infection. In this experiment, serum samples from patients infected with *K. pneumoniae* were collected. (**A**) Reciprocal of FimA-specific antisera dilution of the human convalescent sera from *K. pneumoniae* infection. OVA_192–201_ peptide-specific antisera were used as a negative control. (**B**) The mean titer of FimA-specific IgG antibodies in human sera was 1:14,720, which was significantly different from that of the OVA_192–201_ peptide group (****, *p* < 0.0001). (**C**) The levels of each subtype of antibodies in the antisera of mice immunized with the single peptide-KLH or mixed peptides-KLH. (**D**) Fine mapping of B-cell immunodominant epitopes of FimA non-human convalescent sera after *K. pneumoniae* infection. The four novel immunodominant epitopes, including FimA_97–114_ (*, *p* = 0.0257), FimA_103–120_ (**, *p* = 0.0092), FimA_109–126_ (**, *p* = 0.0084), and FimA_145–160_ (****, *p* < 0.0001), identified in the FimA immunized mice were also found to be immunodominant in the human convalescent sera. (**E**) All overlapping peptides were used to map the linear B-cell epitopes recognized by human serum samples. In the antibody of human convalescent sera, there were also three other positive immunodominant epitopes, namely FimA_1–18_ (**, *p* = 0.0087), FimA_49–60_ (***, *p* = 0.0009), and FimA_55–72_ (***, *p* = 0.0009).

In this experiment, serum samples from patients infected with *K. pneumoniae* exhibited antigen–antibody reactivity with FimA, with an average FimA-specific IgG antibody titer of 1:14,720 (Figure 6A,B), and IgG1 was the predominant isotype (Figure 6C). Using 18aa overlapping peptides coated with FimA, the immunodominant response epitopes were detected in the infection sera mentioned above. As shown in Figure 6D, with human sera, the four novel immunodominant epitopes including FimA_97–114_ (*p* = 0.0257), FimA_103–120_ (*p* = 0.0092), FimA_109–126_ (*p* = 0.0084), and FimA_145–160_ (*p* < 0.0001) identified in the FimA immunized mice were also found to be immunodominant in the human convalescent sera. Additionally, in the human convalescent sera, there were also three other positive immunodominant epitopes, namely FimA_1–18_ (*p* = 0.0087), FimA_49–60_ (*p* = 0.0009), and FimA_55–72_ (*p* = 0.0009) (Figure 6E).

## 4. Discussion

*K. pneumoniae* is capable of causing severe and life-threatening infections such as pneumonia, bacteremia, and complicated urinary tract infections [20]. The emergence of hypervirulent and antibiotic-resistant *K. pneumoniae*, especially carbapenem resistance, is worrisome and requires effective methods for treatment. This prevalence varies widely across geographic regions, ranging from 12% to 45% in Asian hypervirulent *K. pneumoniae*-endemic areas [21]. Due to its resistance to nearly all antimicrobials, the development of alternative therapies like antibodies and vaccines is urgently needed to overcome antibiotic resistance and reduce the health and economic impacts of diseases caused by this pathogen. Despite the implementation of antibiotics, globally, *K. pneumoniae* prevalence is stable. It is undeniable that vaccines to prevent and control *K. pneumoniae* prevalence by active or passive immunization are needed [22].

The most successful prophylactic vaccines are based on the induction of neutralizing antibodies. *K. pneumoniae* uses a range of virulence factors to ensure its survival and promote disease development; these factors include capsule, types I and III fimbriae, and the type VI secretion system. As the major structural and adhesive component of type I fimbriae, FimA mediates the initial, critical step of bacterial attachment to host epithelial cells, thereby facilitating colonization and subsequent invasion of *K. pneumoniae* [23,24]. Our data corroborate the immunogenic potential of FimA, as active immunization with the full-length protein conferred a remarkable 90% survival rate against a lethal dose of the ATCC700721. Our findings demonstrate that FimA is a potent immunogen that elicits robust protective immunity against lethal challenge with *K. pneumoniae*.

Antibody responses play a key role in fighting against invasive *K*. *pneumoniae* infection [25]. In this study, the titers of FimA-specific antibodies in mice from the FimA + Al(OH)_3_ group were significantly higher than those in the PBS + Al(OH)_3_ group (*p* < 0.0001). This protection was associated with a robust IgG1 antibody response, underscoring the importance of humoral immunity in mediating protection against this pathogen. This aligns with previous studies highlighting FimA-specific antibody-mediated prophylactic protection as a key defense mechanism against *K. pneumoniae* infection [26].

While whole-protein vaccines are effective, they can sometimes induce non-protective or even deleterious immune responses [27]. Immunodominance defines the hierarchical immune response to competing antigens in complex immunogens [28]. Epitope-based vaccines offer a refined alternative, focusing the immune response on critical, protective determinants, thereby enhancing safety and efficacy in infectious diseases [29,30]. However, little is known regarding B-cell immunodominance of FimA despite its importance in immunity to *K. pneumoniae*. In this context, we employed a peptide-based overlapping ELISA approach to deconvolute the antibody response to FimA and successfully mapped four immunodominant linear B-cell epitopes: FimA_97–114_, FimA_103–120_, FimA_109–126_, and FimA_145–160_. All these were novel epitopes of FimA. To the best of our knowledge, these represent the first reported linear immunodominant B-cell epitopes for the FimA protein, providing a valuable resource for vaccine development and serodiagnosis applications. Further experiments revealed that these epitopes induced robust antibody responses, mainly an IgG1 response.

A significant and intriguing finding was the correlation between epitope location and immunogenicity. Three of the four identified epitopes (FimA_97–114_, FimA_103–120_, and FimA_109–126_) are situated within the predicted α-helical region of the FimA protein, while the fourth (FimA_145–160_) resides in a β-sheet domain. Notably, the α-helical epitopes, particularly FimA109–126, elicited the highest titers of epitope-specific antisera. This observation is consistent with the notion that α-helical structures, often found on the surface of proteins, are more accessible to antibodies and can serve as potent B-cell epitopes. The structural context of these epitopes is crucial for their recognition by B-cell receptors, and the dominance of α-helical epitopes in this study suggests that this region of FimA is a particularly attractive target for vaccine design.

The potential of these epitopes is significantly bolstered by their exceptional conservation. Our analysis revealed 100% sequence identity for these epitopes across 30 diverse *K. pneumoniae* clinical strains. This high degree of conservation is a critical attribute for the *K. pneumoniae* vaccine candidate, as it suggests the potential for broad protection against a wide range of clinical isolates. The subsequent challenge experiments using a mixture of these four epitope-KLH conjugates partially validated this hypothesis. Immunization with the mixed peptides provided cross-protection against four different clinical isolates (10CYZ, 13LGY, 19ZXQ, and 22CZY), albeit with varying survival rates (40–60%). This level of protection against heterologous strains, which likely possess distinct genetic backgrounds as determined by MLST, underscores the potential of this multi-epitope approach to confer broad-spectrum immunity.

Interestingly, the mixed-epitope formulation did not achieve the same level of protection (90%) as the full-length FimA protein against the standard strain. This discrepancy can be attributed to several factors. First, the full-length protein may present conformational epitopes or a broader repertoire of linear epitopes that contribute to the overall protective response. Second, the process of conjugating peptides to KLH, while necessary for enhancing immunogenicity, may alter the presentation or accessibility of the epitope compared to its native form within the intact protein. Third, the protection observed could be synergistic, involving not only B-cell responses but also T-cell help that is more optimally engaged by the whole protein. The mixed epitopes induced a predominantly IgG1 response, indicative of a Th2-skewed immune response. While IgG1 antibodies are known to be highly effective in infection, a Th1 component, which the whole protein might also elicit, could contribute to more robust and durable protection. Furthermore, although the present study has precisely mapped the B-cell dominant epitopes of FimA, a second round of fine mapping using peptides overlapping by one or two amino acids within the key regions has not yet been performed to determine the minimal essential binding sequences. This should be addressed in future studies.

In conclusion, this study provides a comprehensive immunological characterization of the *K. pneumoniae* FimA protein. We have identified four novel, highly conserved, immunodominant linear B-cell epitopes that elicit protective antibody responses. The differential immunogenicity of these epitopes, particularly those within the α-helical region, provides key insights for vaccine design. Our results demonstrate that a multi-epitope formulation can confer cross-protection against diverse clinical isolates, representing a significant step forward in the development of a broadly protective, epitope-based vaccine against *K. pneumoniae*. Future efforts will focus on optimizing the delivery and formulation of these epitopes, perhaps using a multi-antigenic platform, to enhance their immunogenicity and broaden the protective efficacy.

## Data Availability

The original contributions presented in the study are included in the article. Further inquiries can be directed to the corresponding authors.
